# Vegetable waste-based diet supplemented with phytogenic feed additives improves growth performance, carcass characteristics, and economic efficiency in broilers under varying energy densities

**DOI:** 10.3389/fvets.2025.1688247

**Published:** 2025-12-17

**Authors:** Jalmeen Kour, Shivani Katoch, Varun Sankhyan, Madhu Suman, Abhishek Dhiman, Nidhi Daroch, Shubhani Sharma, Kshitij Katkar

**Affiliations:** Chaudhary Sarwan Kumar Himachal Pradesh Krishi Vishvavidyalaya, Palampur, India

**Keywords:** turmeric powder, cinnamon extract, nutrient digestibility, metabolizable energy, *Urtica dioica* (nettle), broilers

## Abstract

**Background:**

The rising cost of conventional feed ingredients and environmental concerns related to agro-waste disposal have created a need for sustainable feed alternatives. Vegetable waste provides valuable nutrients but contains anti-nutritional factors that may limit utilization. Strategic supplementation with small quantities of phytogenic additives such as cinnamon extract and turmeric powder may enhance nutrient utilization and overall performance.

**Aim:**

This study evaluated the effects of partial substitution of conventional feed ingredients with vegetable waste, supplemented with low levels of cinnamon extract and turmeric powder, on growth performance, nutrient digestibility, carcass traits, and economic efficiency in broilers.

**Methodology:**

A total of 150 day-old Vencobb-400 broiler chicks were randomly allocated to three dietary treatments for 42 days: T_0_ (control, basal diet), T_1_ (15% vegetable waste−4% *Urtica dioica*, 3% cauliflower, 3% pea, and 5% radish leaves–with 0.1% cinnamon extract and 0.3% turmeric powder), and T_2_ (same as T_1_ with approximately 10% reduced metabolizable energy).

**Results:**

T_1_ demonstrated a significantly greater weight gain (*P* < 0.0001), showing a 16.96% increase over T_0_ and an improved feed conversion ratio of 1.72 over 2.05. Crude protein digestibility increased from 84.3% in T_0_ to 89.7% in T_1_. T_1_ also achieved the highest carcass yield, gross profit (40.0%) and European Efficiency Factor (321.96). No adverse effects were observed on liver enzyme levels.

**Conclusion:**

Incorporating 15% vegetable waste, supported by minimal phytogenic supplementation, significantly improves broiler performance and profitability. Future research should explore optimization of waste composition and dietary energy levels for commercial application.

## 1 Introduction

Worldwide poultry production has transformed from traditional backyard operations to a technologically advanced industry. In 2025, global chicken meat production is forecast to reach 105.8 million metric tons (1), representing a 2% increase from the previous year. According to the United Nations Food and Agriculture Organization, pork remains the most widely consumed meat globally (36%), followed by poultry (33%) (2). The rising demand for poultry products, driven by increasing urbanization and income levels, has led to intensified broiler production systems. While this growth contributes positively to food security and rural livelihoods, it also brings forth critical challenges—most notably, the availability and cost of feed. Feed constitutes the largest operational expense in broiler production, accounting for approximately 68–75% of the total cost (3). A major sustainability challenge stems from the broiler industry's heavy reliance on conventional feed ingredients like maize and soybean—commodities that are increasingly in demand for both human consumption and biofuel industries (4). Recent market analyses indicate that the global poultry feed market was valued at approximately USD 233 billion in 2024 and is projected to reach around USD 259 billion by 2030, growing at a CAGR of 1.8%, reflecting the rising global feed costs and economic pressure on poultry producers. Moreover, resource constraints such as land degradation, water scarcity, and ecological pollution from conventional feed production necessitate innovative solutions.

Within this framework, incorporating vegetable waste and by-products unsuitable for human consumption as alternative feed ingredients offer a promising strategy. Globally, an estimated 1.05 billion tons of food was wasted in 2022, generating 8–10% of total greenhouse gas emissions and occupying nearly 30% of agricultural land (5). In countries like India, China, the Philippines, and the United States, vegetable waste alone—which includes leaves, discarded peas, and other non-edible parts—totals an estimated 55 million tons annually (6). This growing dependence raises serious sustainability concerns for broiler production, including competition with human food supplies, land and water scarcity, and the environmental impacts of large-scale feed production (7). In this context, identifying and utilizing alternative feed sources such as non-edible vegetable wastes is essential to improve sustainability and reduce environmental pollution. Globally, 10–20% of horticultural produce is discarded, representing a significant waste of resources. Redirecting this surplus vegetable biomass into broiler diets provides a dual benefit: reducing feed costs and mitigating ecological impacts. Broilers, owing to their efficient digestive and metabolic systems, can effectively convert vegetable waste into high-quality animal protein. Recent studies suggest that moderate dietary fiber levels (2–3%) can improve gizzard development and function in broilers by providing mechanical stimulation that enhances grinding activity and digestion efficiency (8). Additionally, components like *Urtica dioica* offer further nutritional benefits. It contains a wide range of bioactive compounds, including flavonoids, polysaccharides, isolectins, terpenes, sterols, proteins, vitamins, minerals, volatile compounds, and fatty acids such as stearic, oleic, palmitic, and linolenic acids. The plant's leaves are rich in calcium, phosphorus, potassium, sodium, magnesium, iron, and vitamins B, C, and K. It also contains carotenoid, chlorophyll, glucokinins, and essential amino acids, which may support overall health and nutrient utilization in poultry (9).

Vegetable-derived feed materials, though nutrient-rich, often contain anti-nutritional compounds—such as phytic acid, nitrates, oxalates, tannins, alkaloids, trypsin inhibitors, and α-galactosides—that may hinder nutrient absorption and growth performance. At the molecular level, dietary modulation has been shown to influence intestinal immune signaling and epithelial integrity, thereby regulating nutrient absorption and host defense mechanisms in monogastric animals (10). To counteract the negative effects of these anti-nutrients, phytogenic feed additives (PFA) have emerged as a sustainable strategy. Beyond their antioxidant and anti-inflammatory properties, PFAs and vegetable-derived feed components can beneficially modulate the gut microbiota composition, promoting beneficial microbial taxa and maintaining intestinal ecological balance, as revealed by recent metagenomic studies (11). PFAs have also been shown to enhance intestinal morphology, digestive enzyme secretion, and mucosal immunity through antioxidant and anti-inflammatory pathways, thereby improving gut barrier integrity and nutrient assimilation in broilers (12). Moreover, phytogenic and botanical feed additives exhibit immunomodulatory effects by enhancing cytokine expression, stimulating macrophage activity, and promoting both innate and adaptive immune responses, thereby supporting overall health and resilience in broilers (13). PFA are known to improve nutrient digestibility, immune function, and growth performance while also meeting consumer demand for antibiotic-free poultry products. The global PFA market was valued at approximately USD 1.05 billion in 2024 and is projected to reach USD 1.48 billion by 2030, growing at a CAGR of 6.04% from 2025 to 2030. The poultry segment accounted for about 44% of this market in 2024, driven by the regular use of PFAs to improve poultry product quality and by ongoing research into novel phytogenic substances (14). Therefore, integrating processed vegetable waste with appropriate PFA not only mitigates anti-nutritional constraints but also lowers feed costs through the use of locally available plant-based by-products, enhancing both sustainability and profitability in broiler production.

Among these, cinnamon (*Cinnamomum zeylanicum*) and turmeric (*Curcuma longa*) are known for their antimicrobial, antioxidant, and immunomodulatory effects. The essential oil of cinnamon bark is rich in trans-cinnamaldehyde and other biologically active compounds such as cinnamyl acetate, eugenol, and β-caryophyllene which contribute to its antimicrobial, antioxidant, and immunomodulatory properties (15). The major active principle in cinnamon is the cinnamaldehyde. Antimicrobial properties of cinnamon are related to its cinnamaldehyde content followed by eugenol and carvacrol content. Curcumin is the key bioactive component responsible for the biological properties of *Curcuma longa* (16). Furthermore, adding turmeric (*Curcuma longa*) to poultry feed could reduce the harmful effects of aflatoxins on the liver and kidneys of chickens (17). Both cinnamon and turmeric have been shown to enhance feed intake, stimulate digestive enzymes, improve nutrient absorption, and support overall broiler performance. Similar findings have been reported where dietary inclusion of cinnamon extract and turmeric powder improved feed conversion efficiency, nutrient utilization, and broiler performance without adverse effects (18, 19). However, limited studies have examined the combined use of vegetable waste-based feed with phytogenic supplements under reduced energy diets. Addressing this gap is essential to develop sustainable and cost-effective feeding strategies without compromising broiler performance. Therefore, the present study was conducted to evaluate the effects of incorporating 15% vegetable waste, supplemented with cinnamon extract and turmeric powder, in broiler diets on growth performance, nutrient digestibility, carcass traits, and economic efficiency. In addition, a group receiving the same vegetable waste and phytogenic additives with approximately 10% reduced metabolizable energy was included to assess the impact of lower dietary energy density. We hypothesized that the vegetable waste-based diet supplemented with phytogenic additives would improve broiler performance and profitability compared to a conventional diet, and that moderate energy reduction may compromise these benefits.

## 2 Materials and methods

### 2.1 Ethical approval

The experiment was approved by the Institutional Animal Ethics Committee (IAEC) at DGCN COVAS, CSKHPKV, Palampur, Himachal Pradesh, India (Proposal No. IAEC/24/07, approved March 16, 2024). Approval covered the use of 150 Vencobb broiler chicks, in compliance with institutional and national guidelines for the care and use of animals.

### 2.2 Feed ingredient collection and preparation

Vegetable waste—including stinging nettle (*Urtica dioica*), cauliflower leaves and stems, radish leaves, and pea pods (with discarded peas)—was collected from local markets and households in Palampur, Himachal Pradesh. The material was washed, blanched at 80 °C for 2–3 min, and cooled. It was then sun-dried for 2 days under ambient conditions (32–35 °C), followed by oven drying at 55 °C for 6 h to ensure uniform moisture removal. The dried material was ground and incorporated into the basal diets along with phytogenic feed additives. The vegetable waste mixture used in the experimental diets was analyzed for proximate and mineral composition. The results are presented in Table 1. This processing method has been reported to reduce heat-labile antinutritional factors such as phytates, tannins, and saponins (20, 21). However, more heat-stable compounds such as lectins and protease inhibitors may not be fully inactivated under these conditions, as their deactivation generally requires higher temperatures and moist heat treatments (21). Roasted soybean was processed separately by dry roasting to improve palatability and reduce anti-nutrient levels. Phytogenic feed additives, including turmeric powder and cinnamon extract, were included to enhance the bioactive profile of the diet. Cinnamon bark (50 g) was extracted in 400 mL of 50:50 ethanol–water for 24 h, filtered, dried at room temperature for 3–4 days, ground, and stored at 4 °C. Other feed ingredients—such as maize, soya flakes, roasted soybean, vegetable oil, sodium bentonite, dicalcium phosphate, limestone, and premixes—were procured from the Department of Animal Nutrition Feed Store, DGCN COVAS, CSKHPKV and analyzed for nutrient composition prior to diet formulation.

**Table 1:** Proximate and mineral composition of vegetable waste mixture (% on DM basis).

**Ingredients**	**DM%**	**CP%**	**CF%**	**EE%**	**Ca%**	**P%**	**TA%**	**AIA%**	**NFE%**	**ME(Kcal/Kg)**
*Urtica dioica*	100.00	29.10	8.55	4.94	4.18	0.12	20.00	2.80	48.82	2,525
Cauliflower waste	100.00	20.30	18.43	2.75	0.16	0.08	17.73	1.20	40.78	2,184
Pea waste	100.00	29.46	18.83	2.48	0.05	0.10	5.02	0.79	44.19	2,578
Radish leaves	100.00	27.01	14.90	3.67	0.89	0.09	17.82	1.85	37.11	2,335

### 2.3 Experimental design

A total of 150 day-old Vencobb-400 chicks were randomly allocated to three treatments, each with five replicates of 10 birds (Table 2). Diets were formulated for pre-starter (0–14 d), starter (15–21 d), and finisher (22–42 d) phases according to ICAR (2013) nutrient specifications. All diets were iso-nitrogenous and iso-caloric, except the reduced-metabolizable energy (ME) treatment. The T_0_ (control) group was fed a standard ICAR-based basal diet. The T_1_ group was fed a diet consisting of 85% basal feed and 15% vegetable waste (4% *Urtica dioica*, 3% cauliflower waste, 3% pea waste, 5% radish leaves), supplemented with 0.1% cinnamon extract and 0.3% turmeric powder. The T_2_ group was fed the same diet as T_1_, but with approximately 10% lower ME (Table 3). In this study, 15% vegetable waste was included in the broiler diet based on previous research demonstrating that vegetable by-products can be safely incorporated at levels up to 15–20% without adversely affecting growth performance or health (22–24). The inclusion levels of the nutraceuticals were based on previous research. Supplementing broiler diets with 0.1% cinnamon extract significantly enhanced feed conversion efficiency, nutrient utilization, and meat quality (18). Additionally, a previous study showed that turmeric powder, when used up to 0.5%, supported weight gain without causing any adverse effects (19).

**Table 2:** Experimental design.

**Treatment (diet)**	**Number of replicates**	**Broilers per replicate**	**Total animals per treatment**
T_0_	5	10	50
T_1_	5	10	50
T_2_	5	10	50

**Table 3:** Composition and nutrient levels of experimental diets (% DM basis).

**Ingredients**	**Pre-Starter**			**Starter**			**Finisher**		
	**T**_0_	**T**_1_^*^	**T**_2_^*^	**T**_0_	**T**_1_^*^	**T**_2_^*^	**T**_0_	**T**_1_^*^	**T**_2_^*^
Maize	52.00	42.95	42.95	51.00	44.002	45.00	57.00	49.00	50.00
Roasted Soybean	19.00	22.00	22.00	21.00	25.00	23.00	26.00	25.00	20.00
Soya Flakes	22.50	13.50	13.50	21.00	9.00	10.00	10.00	4.00	8.00
Vegetable Oil	3.00	3.00	0.00	3.00	3.00	0.00	3.00	3.00	0.00
*Urtica dioica*	0.00	4.00	4.00	0.00	4.00	4.00	0.00	4.00	4.00
Cauliflower Waste	0.00	3.00	3.00	0.00	3.00	3.00	0.00	3.00	3.00
Pea waste	0.00	3.00	3.00	0.00	3.00	3.00	0.00	3.00	3.00
Raddish Leaves waste	0.00	5.00	5.00	0.00	5.00	5.00	0.00	5.00	5.00
Premix^**^	1.00	1.00	1.00	1.00	1.00	1.00	1.00	1.00	1.00
Dicalcium phosphate	1.20	1.20	1.20	1.50	1.50	1.50	1.50	1.50	1.50
Limestone Powder	1.35	1.35	1.35	1.50	1.50	1.50	1.50	1.50	1.50
Sodium Bentonite	0.00	0.00	3.00	0.00	0.00	3.00	0.00	0.00	3.00
Total	100	100	100	100	100	100	100	100	100
**Analyzed**									
Crude protein (%)	22.07	22.52	22.52	21.97	21.93	21.60	19.83	20.10	19.90
Crude fiber (%)	4.07	5.54	5.54	4.02	5.41	5.38	3.63	5.14	5.15
Ether Extract (%)	6.51	7.36	7.36	6.93	8.05	7.60	8.15	8.12	7.00
Nitrogen-Free Extract (%)	51.27	47.82	47.82	50.13	47.52	48.30	51.63	49.55	50.89
Total Ash (%)	3.06	4.75	4.75	3.00	4.53	4.52	2.36	4.15	4.25
Calcium (%)	1.01	1.15	1.15	1.16	1.30	1.30	1.13	1.28	1.29
Phosphorus (%)	0.57	0.51	0.51	0.62	0.55	0.55	0.58	0.53	0.54
**Calculated**									
ME(KCal/Kg)	3088.67	3051.88	2781.88	3082.10	3079.82	2791.19	3166.50	3102.10	2778.80
Lysine (%)	1.20	1.20	1.20	1.09	1.10	1.10	0.94	0.94	0.94
Methionine (%)	0.52	0.52	0.52	0.48	0.48	0.48	0.41	0.41	0.41

^*^(Turmeric powder 300 g/100 Kg + Cinnamon extract 100 g/100 Kg).

^**^Traymix = 10 g (Vitamin A−82,500 IU, Vitamin B2−50 mg, Vitamin D3−12,000 IU, Vitamin K−10 mg/g); Ventribeeplus = 25 g (Vitamin B1−25 mg, B6−35 mg, B12−250 μg, Vitamin E−225 mg, Pantothenate−225 mg, Niacinamide−300 mg, Folic acid−20 mg/5 g); Nutri-Choline = 90 g (Choline Chloride−60% per kg); E-Care Se Super Forte = 10 g (Vitamin E−500 g/kg, Selenium−1,000 mg/kg); Trace Minerals = 100 g (Manganese Sulfate−20 g, Ferrous Sulfate−40 g, Ferrous Oxide−2 g, Zinc Oxide−17.5 g, Copper Sulfate−5 g, Potassium Iodide−2.5 g, Cobalt Chloride−3 g); Phytoplus = 5 g (minimum 5,000 FTU phytase/g); Opti-Zyme-L = 10 g (Xylanase, Cellulase, β-glucanase, Pectinase, α-amylase, Protease, α-galactosidase, β-galactosidase, Lipase, Mannanase); Quinacox = 45 g (containing Diclazuril−1 g/kg); Toxin Binder = 45 g; Lysine and Methionine added to balance dietary requirements.

### 2.4 Housing and management

From day 0–7, chicks were housed in battery brooders (36 × 72 inches) at 33–35 °C and 50–60% relative humidity. From day 8–42, they were reared in deep-litter pens (5 × 4 × 6 ft), stocking density of approximately 5.4 birds/m^2^, with 6 cm sawdust bedding at a temperature of 17.4–25 °C and 40–60% humidity. Feed and water were provided *ad libitum*, with 24-h lighting and adequate ventilation. Daily feed intake was recorded per replicate.

### 2.5 Analytical procedures

Proximate composition [dry matter (DM), crude protein (CP), ether extract (EE), crude fiber (CF), total ash (TA), and nitrogen-free extract (NFE)] of feed ingredients, experimental diets, and feces was analyzed according to AOAC methods (25). DM was determined by oven drying; TA by muffle furnace; EE by Soxhlet extraction; CP by the Kjeldahl method; CF using a fiber analyzer. Calcium (Ca) was estimated by atomic absorption spectrophotometry, and phosphorus (P) by the spectrophotometric method (26). The ME of ingredients was calculated using a previously suggested equation (27).

### 2.6 Assessment of apparent nutrient digestibility

On day 35, a metabolic trial was conducted using the total fecal collection method. Two birds per replicate were placed in battery brooders for a 3-day adaptation, followed by 5 days of fecal collection. Feed was offered twice daily, and refusals were weighed to determine intake. Feces were pooled per replicate, with one portion preserved in 5% sulfuric acid for nitrogen estimation and the other for DM estimation. Digestibility coefficients for DM, CP, CF, EE, Ca, and P were calculated using the equation:


$$Digestibility\left(\%\right)=\frac{Nutrient\;Intake-Nutrient\;Outgo\;in\;feces}{NutrientIntake}\times 100$$

where nutrient intake is the amount of a given nutrient consumed by the birds and nutrient outgo is the amount excreted in feces during the collection period.

### 2.7 Growth performance

Initial body weight was measured before the chicks were housed, followed by weekly measurements. Weight gain was calculated as the difference between final and initial body weight for each replicate. Feed intake was monitored daily by providing a measured quantity of feed to each replicate and recording the feed refusal at the end of the day. Based on the collected data, feed conversion ratio (FCR: total feed consumed during the period divided by total weight gain) was calculated for each growth phase and the overall period.

### 2.8 Carcass characteristics and gizzard thickness

At day 42, two birds per replicate (average body weight) were fasted overnight and slaughtered by the jhatka method. Standard dressing procedures were followed, and weights of carcass components were recorded. Gizzard wall thickness was measured at three external points using a vernier caliper, and the mean was calculated per bird.

### 2.9 Meat quality assessment

Meat samples were collected post-slaughter and stored at −20 °C. Proximate composition was determined per AOAC methods (25). Sensory evaluation followed the described method (28): thawing at 3 °C for 18 h, deboning, cubing (2 × 2 × 2 cm), salting (1% NaCl), and pressure cooking to 80 ± 2 °C for 15 min. Sensory evaluation was conducted by panelists, comprising faculty and laboratory staff from the Department of Livestock Products Technology, Animal Nutrition, CSKHPKV, Palampur, who had prior familiarity with meat quality assessment but were not formally trained in sensory discrimination protocols. They scored flavor, texture, tenderness, juiciness, appearance, and overall acceptability on an 8-point scale (8 = like extremely; 1 = dislike extremely).

### 2.10 Serum biochemical analysis

On day 42, blood samples were collected from two birds per replicate (10 birds per treatment) via jugular venipuncture using sterile 24-gauge needles. Samples were transferred into clot activator vials for serum separation. Serum was obtained by allowing samples to clot for 3–4 h at room temperature, followed by centrifugation at 2,000 rpm for 15 min. ALT (SGPT) and AST (SGOT) activities were determined using commercial diagnostic kits (LyphoCHEK SGPT and SGOT kits; Agappe Diagnostics Ltd., Kerala, India). For each test, 100 μL of serum and 1,000 μL of reagent were mixed, incubated at 37 °C for 1 min, and the change in absorbance per minute (Δ OD/min) was recorded over 3 min using a MispaViva semi-automated clinical chemistry analyzer (Agappe Diagnostics Ltd.).

### 2.11 Statistical analysis

Data were analyzed using a one-way Analysis of Variance (ANOVA) under a completely randomized design with IBM SPSS v30.0. Treatment means were compared using Tukey's Honest Significant Difference (HSD) test, and differences were considered statistically significant at *P* < 0.05, with additional levels of significance reported as *P* < 0.01 and *P* < 0.0001 where applicable. Results are expressed as mean ± SD (29). In addition, effect sizes (Cohen's *d*) were calculated for the primary performance indicators (overall final body weight, daily weight gain, feed intake, and FCR) to quantify the magnitude of treatment differences and to evaluate the adequacy of the replication level. Cohen's d was calculated for pairwise comparisons against the control (*T*_0_) using:


$$d=\frac{M_{t}-M_{c}}{S_{pooled}}$$

$$S_{pooled\;}=\sqrt{\frac{{SD_{t}}^{2}+{SD_{c}}^{2}}{2}}$$

Standard deviations (SD) were obtained from the reported SEM using:


$$SD=SEM\times \sqrt{5}$$

The exact Cohen's *d* values were:

Final body weight: T_1_ vs. T_0_ = 5.22; T_2_ vs. T_0_ = 1.54

Weight gain: T_1_ vs. T_0_ = 5.30; T_2_ vs. T_0_ = 1.52

Feed intake: T_1_ vs. T_0_ = 1.07; T_2_ vs. T_0_ = 1.88

FCR: T_1_ vs. T_0_ = 4.85; T_2_ vs. T_0_ = 1.57.

All effect sizes exceeded the conventional threshold for a large effect (*d* ≥ 0.8), with several reaching very large to extremely large magnitudes (*d* = 1.07–5.30), indicating that five replicates per treatment were adequate to detect biologically meaningful differences.

### 2.12 Economic evaluation

Economic performance was assessed using viability percentage, the European Efficiency Factor (EEF), and the cost of producing 1 kg live weight (Cm).

Viability (%) represents the proportion of broilers that survived until the end of the trial and was calculated as (30):


$$Viability\;\left(\%\right)\;=\frac{No.\;of\;viable\;birds}{Total\;birds}\times 100$$

The EEF, a composite index of growth rate, feed efficiency, survival rate, and rearing duration, was calculated as:


$$EEF=\frac{BW\times Viability\left(\%\right)}{FCR\times A}\times 100$$

where BW is the average body weight (kg), FCR is the feed conversion ratio, and A is the age of broilers (31). The production cost per kilogram of live weight was determined as:


$$C_{m}=FCR\times C_{f}$$

where, *C*_*m*_ is the production cost (INR) per kg live weight and C_f_ is the cost of feed per kg (32).

## 3 Results

### 3.1 Growth performance

Growth performance was recorded during pre-starter (0–14 days), starter (15–21 days), finisher (22–42 days), and overall (0–42 days) phases (Table 4). Initial body weight values across all treatment groups were highly uniform. The highest final body weight was observed in T_1_ compared to T_0_ (control), while T_2_ (same diet as T_1_ with approximately 10% reduced ME) recorded lowest body weight during all the phases.

**Table 4:** Growth performance of broilers fed experimental diets during different phases: pre-starter (0–14 d), Starter (15–21 d), Finisher (22–42 d), and Overall (0–42 d).

**Treatment**	**T_0_**	**T_1_**	**T_2_**	***p*-value**
**Final body weight (g)**				
Pre-Starter (0–14 days)	232.10 ±6.97^a^	245.74 ±6.12^a^	214.92 ±3.21^b^	< 0.01
Starter (15–21 days)	600.42 ±10.14^ab^	631.32 ±7.68^b^	545.88 ±5.71^a^	< 0.0001
Finisher (22–42 days)	2044.0 ± 55.4^a^	2382.6 ± 70.8^b^	1969.7 ± 40.1^a^	< 0.001
Overall (0–42 days)	2046.0 ±25.67^b^	2382.6 ±31.67^c^	1969.7 ±17.93^a^	< 0.0001
**Weight gain per day (g)**				
Pre-Starter (0–14 days)	13.39 ±0.49^ab^	14.36 ±0.46^b^	12.17 ±0.22^a^	< 0.01
Starter (15–21 days)	52.62 ± 1.57^b^	55.08 ± 3.63^b^	47.28 ± 1.70^a^	< 0.01
Finisher (22–42 days)	68.74 ± 3.43^a^	83.40 ± 3.63^b^	67.80 ± 1.60^a^	< 0.0001
Overall (0–42 days)	47.60 ±0.59^b^	55.67 ±0.76^c^	45.84 ±0.43^a^	< 0.0001
**Feed intake per day (g)**				
Pre-Starter (0–14 days)	27.26 ±0.08^c^	26.89 ±0.11^b^	26.14 ±0.07^a^	< 0.0001
Starter (15–21 days)	97.75 ± 0.70^c^	95.65 ± 0.11^b^	94.82 ± 0.28^a^	< 0.0001
Finisher (22–42 days)	144.28 ± 0.26^a^	145.48 ± 0.15^b^	146.42 ± 0.14^c^	< 0.0001
Overall (0–42 days)	97.52 ± 0.05^a^	97.64 ± 0.05^ab^	97.73 ± 0.05^b^	< 0.05
**Feed conversion ratio**				
Pre-Starter (0–14 days)	2.067 ±0.076^a^	1.898 ±0.067^a^	2.201 ±0.042^b^	< 0.05
Starter (15–21 days)	2.015 ± 0.087^b^	1.772 ± 0.055^a^	2.114 ± 0.033^b^	< 0.01
Finisher (22–42 days)	2.117 ± 0.112^b^	1.756 ± 0.077^a^	2.175 ± 0.052^b^	< 0.0001
Overall (0–42 days)	2.051 ± 0.029^b^	1.720 ± 0.032^a^	2.140 ± 0.021^b^	< 0.0001

Different superscripts (a, b, c) within a row indicate statistically significant differences among treatments. Overall *p*-values for each parameter are reported in the table, ranging from *p* < 0.05 to *p* < 0.0001.

A similar trend was observed in daily weight gain, with T_1_ showing significantly higher daily gains than both T_0_ (control) and T_2_ (*p* < 0.01). T_1_ consistently exhibited superior daily weight gain across all phases. Despite receiving the same feed formulation as T_1_, the reduced ME content in T_2_ led to significantly lower daily weight gains in all phases.

During the pre-starter and starter phases, daily feed intake was significantly higher in T_0_ compared to T_1_, while T_2_ had the lowest daily intake (*p* < 0.0001). This trend reversed in the finisher and overall phases, with T_2_ exhibiting the highest daily feed intake, followed by T_1_ and then T_0_. The treatment trends for total feed intake and weight gain mirrored their respective daily values.

Feed conversion ratio (FCR) was significantly improved in the T_1_ group across all phases, while T_2_ exhibited the poorest FCR. T_0_ displayed intermediate efficiency. No mortality was observed in any treatment group throughout the experimental period.

### 3.2 Apparent nutrient digestibility

Apparent digestibility coefficients of proximate nutrients (% DM basis) were determined in broiler chickens (Table 5). T_1_ and T_2_, both supplemented with vegetable waste, exhibited significantly higher digestibility of crude protein (CP) (*p* < 0.05), ether extract (EE), and phosphorus (P) (*p* < 0.01) compared to T_0_ (control). Although CP and P digestibility were statistically similar between T_1_ and T_2_, T_1_ exhibited numerically higher CP and *P*-values, while T_2_ showed a slightly higher ether extract digestibility. No significant differences were observed among groups for crude fiber (CF) and calcium (Ca) digestibility.

**Table 5:** Apparent digestibility coefficients of proximate nutrients (% DM basis) in broiler chickens during metabolic trial.

**Treatment**	**DM**	**CP**	**CF**	**EE**	**Ca**	**P**
**T**_**0**_	61.75 ± 0.96	84.33 ± 0.68^a^	54.52 ± 2.85	83.61 ± 1.76^a^	43.00 ± 1.60	31.60 ± 2.15^a^
**T**_**1**_	63.58 ± 2.69	89.67 ± 2.19^b^	66.49 ± 3.87	88.50 ± 1.47^b^	47.74 ± 3.71	45.77 ± 1.37^b^
**T**_**2**_	62.52 ± 1.15	88.94 ± 0.93^b^	60.90 ± 2.93	90.39 ± 0.78^b^	45.52 ± 1.77	42.53 ± 1.92^b^
**p-Value**	0.772 (NS)	< 0.05^*^	0.068 (NS)	< 0.01^**^	0.445 (NS)	< 0.01^**^

Means bearing different superscripts within a column differ significantly (^*^P < 0.05, ^**^P < 0.01).

### 3.3 Carcass characteristics

#### 3.3.1 Edible by-product yields (% live weight)

The edible by-product yields of broilers under different dietary treatments are presented in Table 6. Dressing percentage (relative to live weight) was significantly higher in T_1_ than in both T_0_ and T_2_ (*p* < 0.01). T_2_ performed similarly to T_0_, suggesting that the approximately 10% reduction in ME did not adversely affect dressing yield. Liver and gizzard yields (as percentages of live weight) were also significantly higher in T_1_ compared to T_0_ (*p* < 0.01), while T_2_ showed the lowest gizzard yield. Heart and skin yields did not differ significantly among treatments.

**Table 6:** Carcass attributes and edible by-product yields (% of live weight) of broiler chickens fed experimental diets.

**Treatment**	**Live weight (gm)**	**Dressed weight (gm)**	**Dressing (%)**	**Giblet**			**Skin (%)**
				**Heart (%)**	**Liver (%)**	**sGizzard (%)**	
**T**_**0**_	1992.50 ± 22.87	1252.50 ± 14.06	62.87 ± 0.18^a^	0.62 ± 0.01	2.19 ± 0.02^a^	2.19 ± 0.04^a^	6.90 ± 0.08
**T**_**1**_	2436.10 ± 19.01	1664.00 ± 16.52	68.30 ± 0.33^b^	0.63 ± 0.00	2.31 ± 0.03^b^	2.42 ± 0.03^b^	6.97 ± 0.05
**T**_**2**_	1937.90 ± 35.22	1226.80 ± 20.09	63.33 ± 0.26^a^	0.61 ± 0.01	2.21 ± 0.02^a^	2.07 ± 0.07^a^	6.82 ± 0.08
***p*** **-value**			< 0.01^**^	0.208 (NS)	< 0.01^**^	< 0.01^**^	0.335 (NS)

Mean bearing different superscript within column differ significantly (^**^P < 0.01).

#### 3.3.2 Gizzard thickness and inedible by-product yields (% live weight)

The data on gizzard thickness and inedible by-product yields are shown in Table 7. Gizzard thickness was significantly higher in T_1_ compared to T_0_ (*p* < 0.01). The highest abdominal fat percentage was recorded in T_0_ (control) group compared to treatments (T_1_ and T_2_). Head yield was significantly higher in T_1_ than in T_0_ (*p* < 0.01), while feet yield did not differ significantly but was numerically higher in T_1_.

**Table 7:** Gizzard thickness and inedible by-product yields (% of live weight) of broiler chickens fed experimental diets.

**Treatment**	**Gizzard thickness**	**Abdominal fat**	**Feet**	**Head**
	**mm**	**%**	**%**	**%**
**T**_**0**_	15.34 ± 0.29^a^	2.02 ± 0.09^b^	4.01 ± 0.04	2.49 ± 0.02^a^
**T**_**1**_	24.62 ± 0.45^c^	1.51 ± 0.02^a^	4.11 ± 0.06	2.91 ± 0.08^b^
**T**_**2**_	16.50 ± 0.42^b^	1.41 ± 0.03^a^	4.07 ± 0.07	2.54 ± 0.01^a^
***p*** **-value**	< 0.01^**^	< 0.01^**^	0.513 (NS)	< 0.01^**^

Mean bearing different superscript within column differ significantly (^**^P < 0.01).

### 3.4 Muscle yields

#### 3.4.1 Breast muscle yield

The breast muscle yields are presented in Table 8. Boned and deboned breast muscle yields were significantly higher in T_1_ compared to both T_0_ and T_2_ (*p* < 0.01), while T_0_ and T_2_ displayed similar values. The deboned percentage of pectoralis major was significantly higher (*p* < 0.01) in T_1_ compared to the control group (*p* < 0.01), whereas pectoralis minor yield showed no significant differences, although numerically higher values were observed in T_1_ and T_2_ than in T_0_.

**Table 8:** Breast muscle yields (% of live weight) of broiler chickens fed experimental diets.

**Treatment**	**With bone (%)**	**Deboned (%)**		**Total deboned (%)**
		**Pectoralis major**	**Pectoralis minor**	
**T**_**0**_	33.14 ± 0.61^a^	18.01 ± 0.68^a^	5.20 ± 0.09	23.21 ± 0.67^a^
**T**_**1**_	36.57 ± 0.20^b^	21.15 ± 0.21^b^	5.31 ± 0.05	24.46 ± 0.22^b^
**T**_**2**_	34.28 ± 0.96^a^	18.96 ± 0.94^a^	5.41 ± 0.18	24.37 ± 0.95^a^
***p*** **-value**	< 0.01^**^	< 0.01^**^	0.474 (NS)	< 0.01^**^

Mean bearing different superscript within column differ significantly (^**^P < 0.01).

#### 3.4.2 Leg muscle yield

The leg muscle yields are shown in Table 9. Thigh muscle yield did not differ significantly among the treatment groups (*p* = 0.123), indicating that dietary modifications did not affect thigh growth. However, drumstick percentage and total leg muscle yield were significantly higher in T_1_ compared to T_0_ (*p* < 0.01), with T_2_ showing intermediate performance. These findings suggest that reduced energy intake in T_2_ may have slightly limited leg muscle deposition.

**Table 9:** Leg muscle yields (% of live weight) of broiler chickens fed experimental diets.

**Treatment**	**Thigh (%)**	**Drumstick (%)**	**Total yield (%)**
**T**_**0**_	9.86 ± 0.10	7.45 ± 0.05^a^	17.30 ± 0.13^a^
**T**_**1**_	10.05 ± 0.04	7.71 ± 0.05^b^	17.75 ± 0.07^b^
**T**_**2**_	9.83 ± 0.08	7.68 ± 0.06^b^	17.51 ± 0.11^ab^
***p*** **-value**	0.123 (NS)	< 0.01^**^	< 0.01^**^

Mean bearing different superscript within column differ significantly (^**^P < 0.01).

### 3.5 Meat quality

#### 3.5.1 Proximate composition

The proximate composition of broiler meat is summarized in Table 10. DM content was significantly higher (*p* < 0.01) in T_1_ compared to the control group (T_0_), while T_2_ and T_0_ recorded similar values. CP content differed significantly among groups (*p* < 0.01), with both T_1_ and T_2_ exhibiting higher CP values than T_0_. The highest (*p* < 0.01) EE content was observed in T_0_ (control), while T_2_ recorded the lowest value (*p* < 0.01). TA content did not differ significantly among treatments, although numerically higher values were recorded in T_1_ compared to T_0_. A highly significant difference (*p* < 0.01) in acid-insoluble ash (AIA) content was observed among groups, with both T_1_ and T_2_ showing higher values than the control (T_0_).

**Table 10:** Proximate Composition of Breast Meat from broilers fed experimental diets.

**Treatment**	**DM (%)**	**CP (%)**	**EE (%)**	**TA (%)**	**AIA (%)**
**T**_**0**_	26.13 ± 0.21^a^	72.59 ± 0.39^a^	5.39 ± 0.15^c^	5.00 ± 0.23	0.33 ± 0.01^a^
**T**_**1**_	28.24 ± 0.28^b^	79.00 ± 1.57^b^	3.85 ± 0.13^b^	5.35 ± 0.12	0.40 ± 0.00^c^
**T**_**2**_	26.03 ± 0.21^a^	77.24 ± 1.56^b^	3.00 ± 0.17^a^	5.20 ± 0.17	0.37 ± 0.01^b^
***p*** **-value**	< 0.01^**^	< 0.01^**^	< 0.01^**^	0.410 (NS)	< 0.01^**^

Mean bearing different superscript within column differ significantly (^**^P < 0.01).

#### 3.5.2 Sensory evaluation

The results of the sensory evaluation are given in Table 11. Differences in sensory attributes among the groups were non-significant for appearance/color and texture/tenderness scores, though numerically higher values were recorded in T_1_ compared to the T_0_ (control), while T_2_ had the lowest scores. The highest scores for flavor and juiciness (*p* < 0.05) were exhibited in treatment groups T_1_ and T_2_ compared to T_0_ (control). The overall acceptability scores were non-significant among the groups; however, numerically, the highest value was recorded in T_1_ compared to T_0_ (control).

**Table 11:** Sensory evaluation scores of broiler meat.

**Treatment**	**Appearance/color**	**Flavor**	**Texture/tenderness**	**Juiciness**	**Overall acceptability**
**T**_**0**_	6.40 ± 0.16	6.70 ± 0.15^a^	6.20 ± 0.13	6.10 ± 0.23^a^	6.80 ± 0.13
**T**_**1**_	6.60 ± 0.16	7.30 ± 0.15^b^	6.30 ± 0.21	7.10 ± 0.23^b^	7.40 ± 0.22
**T**_**2**_	6.10 ± 0.18	6.90 ± 0.18^ab^	6.20 ± 0.20	6.70 ± 0.21^ab^	7.00 ± 0.21
***p*** **-value**	0.128 (NS)	0.043^*^	0.908 (NS)	0.015^*^	0.099 (NS)

Mean bearing different superscript within column differ significantly (^*^P < 0.05).

### 3.6 Liver enzyme profile

The results for liver enzyme profile are given in Table 12. Serum alanine aminotransferase (ALT) and aspartate aminotransferase (AST) levels did not differ significantly among dietary treatments (*p* = 0.473 and 0.973, respectively). ALT values ranged from 24.49 to 27.73 U/L, while AST values ranged from 180.76 to 185.30 U/L, all of which fall within normal physiological limits for broilers.

**Table 12:** Liver enzyme profile across treatment groups.

**Treatment**	**ALT/SGPT (U/L)**	**AST/SGOT (U/L)**
**T**_**0**_	27.73 ± 1.95	185.3 ± 13.37
**T**_**1**_	25.39 ± 2.05	183.03 ± 15.59
**T**_**2**_	24.49 ± 1.71	180.76 ± 11.67
***p*** **-value**	0.473 (NS)	0.973 (NS)

### 3.7 Economic analysis

Economic parameters of different treatment groups are presented in Tables 13, 14. T_1_, included vegetable waste and was supplemented with small amounts of cinnamon extract and turmeric powder, incurred the highest feed cost per bird but also yielded the greatest final body weight and highest sale return, demonstrating its economic advantage. In contrast, T_2_ (with approximately 10% reduced ME) recorded the lowest feed cost but also the lowest return and body weight. The T_0_ (control) group was intermediate in both cost and return.

**Table 13:** Gross profit analysis of broiler production across different dietary treatments.

**Treatment**	**Feed cost (inline2.tif/bird)**	**Final body weight (kg/bird)**	^*^Sale return (inline2.tif)	^**^Profit/loss is sale return-feed cost	**Overall gross profit (%)**
**T**_**0**_	188.15	2.04	367.2	179.05	29.98
**T**_**1**_	189.31	2.38	428.4	239.09	40.03
**T**_**2**_	175.60	1.97	354.6	179.00	29.97

^*^Final BW × Selling price (inline1.tif180/kg live weight); ^**^Sale Return-Feed Cost.

**Table 14:** Economic parameters of broiler production under different dietary treatments.

**Treatment**	**FCR**	**Viability (%)**	**EEF**	**C_m_ (inline2.tif/kg live weight**
**T**_**0**_	2.05	100	236.93	385.70
**T**_**1**_	1.76	100	321.96	333.18
**T**_**2**_	2.14	100	219.18	375.78

EEF, European Efficiency Factor; Cm, Cost of production per kg live weight.

T_1_ achieved the highest gross profit, whereas T_0_ and T_2_ showed comparable results. Although the inclusion of cinnamon extract and turmeric powder slightly increased the feed cost in T_1_, its superior feed conversion ratio (FCR) resulted in a higher gross profit percentage, confirming the economic benefit of the supplementation strategy.

In terms of production efficiency, T_1_ recorded the highest EEF, followed by T_0_, while T_2_ had the lowest EEF due to the energy reduction in its diet. Viability (%) remained 100% across all groups. The cost of production per kilogram of live weight was lowest in T_1_ and highest in T_0_. Collectively, the improved FCR, elevated EEF, and reduced production cost in T_1_ confirm the economic viability of incorporating vegetable waste supplemented with cinnamon extract and turmeric powder in broiler diets

## 4 Discussion

### 4.1 Growth performance

The inclusion of vegetable waste (cauliflower waste, pea waste, radish leaves, nettle) in diet T_1_ significantly improved live weight gain and feed efficiency in broiler chickens. These improvements likely reflect enhanced nutrient digestibility and utilization, arising from the synergistic effects of dietary fiber and PFA (cinnamon and turmeric). Dietary fiber can improve gizzard function and gut motility, facilitating efficient feed passage and nutrient absorption (8). Correlation analysis further supported this relationship, showing a positive association between crude protein digestibility and final body weight (*r* = 0.46), and a negative correlation with FCR (*r* = −0.43), indicating that improved protein utilization contributed to better growth efficiency.

The phytogenic properties of cinnamon and turmeric, attributed to their antimicrobial and antioxidant activities, may also contribute to improved digestion and nutrient utilization (33). Cinnamaldehyde in cinnamon enhances digestion by stimulating salivary secretions and increasing intestinal and pancreatic enzyme activity (34), thereby supporting higher live weight gain. In addition, the antioxidant and immunomodulatory potential of phytogenic compounds such as cinnamaldehyde and curcumin have been shown to mitigate oxidative stress and enhance growth performance in broilers (35). In our study, cinnamon and turmeric supplementation improved FCR, likely due to their digestive and metabolic effects. Similar outcomes were reported in previous studies for cinnamon (36) and turmeric (37) which were attributed to their antioxidant properties and improved protein utilization, supporting growth and feed efficiency in broilers.

In the pre-starter phase, T_1_ achieved a numerically lower FCR compared to the control group (T_0_) despite lower feed intake, although the difference was not statistically significant. T_2_, in contrast, showed poorer FCR, suggesting reduced nutrient utilization efficiency—likely due to its lower energy content. It is important to note that sodium bentonite was used to dilute the diet in T_2_ in order to reduce ME by approximately 10%, replacing energy-contributing ingredients such as maize and soybean meal. While sodium bentonite is commonly used in poultry diets and was selected for its availability and presumed inertness, it may not be entirely metabolically inactive. Previous studies indicate that sodium bentonite can adsorb nutrients and alter intestinal transit and digestibility, which may reduce the bioavailability of some dietary nutrients and thereby affect energy utilization and growth (38). Its nutrient-binding properties could have further reduced the bioavailability of certain nutrients, thereby lowering actual ME more than anticipated. This unintended effect may have contributed to the poorer performance observed in T_2_. This limitation has been acknowledged, and future studies may consider using truly inert diluents such as purified cellulose or acid-washed sand to avoid potential confounding effects. During the starter phase, T_1_ recorded the highest weight gain, whereas T_2_ performed significantly worse (*P* < 0.01), again highlighting the limitations of reduced energy. In the finisher phase, T_2_ improved weight gain compared to earlier phases, accompanied by increased feed intake in both T_1_ and T_2_. This likely reflects compensatory feeding behavior in T_2_ broilers, where intake increased to offset the lower energy density of the feed. Although statistically different (*p* < 0.05), the feed intake values were very close in absolute terms—the differences were minimal (< 0.2 g/day), indicating that palatability and satiety were likely unaffected, and that differences in growth performance were primarily driven by nutrient utilization rather than consumption, as also reported for cinnamon powder supplementation (39).

Therefore, it is plausible that differences in growth performance were driven more by nutrient utilization efficiency than by consumption volume, consistent with previous findings on cinnamon powder supplementation.

Overall, T_1_ consistently demonstrated the most favorable growth outcomes, supporting earlier findings on the benefits of vegetable waste–based diets at moderate inclusion levels (23, 40). However, it is important to note that some studies have reported reduced growth performance at higher levels of vegetable waste inclusion. For example, one study observed significant declines in feed intake and weight gain when vegetable waste inclusion exceeded 25%, indicating potential limitations at excessive levels (41). Similarly, improved live weight and FCR were reported up to 15% inclusion, but performance declined at higher levels (23). These contrasting findings suggest that the effects of vegetable waste depend on the inclusion rate, type and composition of the waste, processing methods, and overall dietary formulation. It is also important to acknowledge that the use of five replicates per treatment may introduce some limitations in the precision of zootechnical performance estimates; however, the treatment differences observed in this study were associated with very large effect sizes (e.g., overall final body weight: *d* = 5.22 for T_1_ vs. T_0_; overall FCR: *d* = 4.85 for T_1vs._ T_0_), indicating that the replication level was sufficient to detect biologically meaningful responses despite the use of only five replicates per treatment.

### 4.2 Nutrient digestibility

DM digestibility remained statistically similar across treatments, indicating that vegetable waste and PFA (turmeric and cinnamon extract) inclusion did not adversely affect overall diet digestibility. CP digestibility was higher in T_1_ and T_2_ compared to the T_0_ (control), suggesting improved protein utilization possibly due to the amino acid profile of vegetable waste or the stimulatory effects of bioactive compounds in turmeric powder or cinnamon extract on digestive enzyme activity (42, 43). In addition, bioactive plant polysaccharides present in these additives may enhance gut barrier integrity and nutrient absorption efficiency by modulating intestinal morphology and microbial balance (44). EE digestibility was significantly higher in T_2_ (*P* < 0.01), likely reflecting adaptive responses to reduced dietary energy, resulting in enhanced lipid mobilization and utilization. This observation aligns with findings that curcumin supplementation can improve lipid digestion (45). Fiber digestibility, while low in all groups as expected for poultry, was slightly higher in T_1_. This improvement may be linked to fermentable fiber in vegetable waste, which promotes short-chain fatty acid (SCFA) production, supporting digestion and nutrient absorption (8, 46). Similar improvements have been reported with *Urtica dioica* supplementation in broiler diets (40).

### 4.3 Carcass characteristics

#### 4.3.1 Edible and inedible by-product yields (including gizzard thickness)

Dressing percentage was significantly higher in vegetable waste-formulated groups, with T_1_ achieving the best values. Notably, T_2_ maintained comparable dressing percentage to T_0_ (control) despite reduced dietary energy, indicating that vegetable waste and PFA can sustain carcass yield even under energy restriction. Similar enhancements in carcass traits and dressing yield have been reported with vegetable waste supplementation (41) and with phytogenics such as turmeric (23, 47) and cinnamon (48).

The highest liver percentage was recorded in T_1_, suggesting enhanced metabolic activity, followed by T_2_, which may reflect physiological adjustments to reduced energy availability. Increases in liver weight have similarly been reported with nettle supplementation, attributed to carvacrol-induced stimulation of pancreatic secretions (49), and with cinnamon and turmeric inclusion in broiler diets (50).

Gizzard weight and thickness were significantly greater in T_1_, followed by T_2_, with T_0_ recording the lowest values. This can be attributed to the high fiber content of vegetable waste stimulating gizzard muscle activity, thereby improving grinding efficiency and nutrient assimilation (8). Similar findings were reported in a study that observed a significant (*p* < 0.05) increase in gizzard thickness with cinnamon extract supplementation (18). Abdominal fat percentage was lowest in T_1_ and T_2_, indicating reduced fat deposition compared to T_0_. This effect may result from the combined lipolytic and antioxidant properties of turmeric and cinnamon bioactives, which enhance fat oxidation, limit lipid accumulation, and simultaneously protect tissues from oxidative stress (47, 51).

### 4.4 Muscle yields

#### 4.4.1 Breast muscle yield

Breast muscle (both deboned and with bone) and pectoralis major yield differed significantly among treatments (*P* < 0.01). T_1_ outperformed both T_0_ and T_2_, indicating that vegetable waste with cinnamon extract, and turmeric powder enhances muscle growth when ME is maintained. T_2_ was statistically similar to T_0_, suggesting that reducing ME by approximately 10% limits muscle development despite supplementation.

The superior breast muscle yield in T_1_ may result from the synergistic effects of vegetable waste fiber-driven digestive improvements and bioactive compound-mediated lipid mobilization from turmeric powder and cinnamon extract, favoring protein deposition. Cinnamon (18, 60), nettle (52), and turmeric (53) have all been shown to enhance carcass quality and muscle growth, supporting the present findings.

#### 4.4.2 Leg muscle yield

Thigh yield remained statistically similar among treatments, indicating that dietary modifications did not significantly influence thigh muscle growth. In contrast, drumstick percentage and total leg muscle yield differed significantly (*P* < 0.01), with T_1_ and T_2_ recording higher values than T_0_. This suggests that vegetable waste, combined with *Urtica dioica*, cinnamon extract, and turmeric powder, enhances muscle deposition by improving nutrient utilization and protein accretion. The ability of alternative feed ingredients to support leg muscle growth without compromising performance aligns with findings on cinnamon supplementation improving thigh muscle yield through enhanced lipid utilization and fatty acid metabolism (16). However, other studies reported no significant effect of cinnamon on carcass traits, indicating that its impact may depend on diet composition and management conditions (33).

Turmeric has also been shown to improve thigh muscle development by promoting nutrient absorption and protein retention (54). The effects of *Urtica dioica* appear inconsistent; supplementation in broiler diets showed no significant impact on thigh yield (52). In the present study, both T_1_ and T_2_ exceeded T_0_ in leg muscle yield despite the reduced energy in T_2_, suggesting that vegetable waste and PFA supplementation can offset moderate energy restriction, though optimal results occur when energy is maintained.

### 4.5 Meat quality

#### 4.5.1 Proximate composition

Breast meat composition showed significant differences in DM, CP, EE, and AIA content, with T_1_ recording the highest CP values. This improvement may be linked to vegetable waste fiber and nettle's role in reducing nitrogen excretion and enhancing protein utilization (52), cinnamon's capacity to improve protein digestion by increasing hydrochloric acid and pepsin secretion (55), and turmeric's reported effects on CP retention, fat deposition, and meat quality (43).

EE content was highest in T_0_ and lowest in T_2_, likely due to reduced energy intake limiting fat deposition. Similar trends were observed in previous work, where cinnamon extract supplementation in low-energy diets resulted in lower meat fat content compared to T_0_ (control) (18). These findings reinforce the concept that higher dietary energy promotes lipid storage, while lower energy intake reduces EE content, possibly through downregulation of lipogenic pathways and upregulation of fatty acid β-oxidation, as reported by Liu et al. (35). Overall, vegetable waste supplemented with turmeric powder and cinnamon extract improved protein retention and reduced fat deposition, enhancing meat quality even under reduced energy conditions. Furthermore, these improvements in carcass and meat quality may also be associated with enhanced intestinal morphology and antioxidant capacity, which together support better nutrient absorption and tissue integrity (56).

#### 4.5.2 Sensory evaluation

Scores for appearance/color, texture/tenderness, and overall acceptability did not differ significantly among groups, though numerically higher values were observed in T_1_ compared to T_0_, with T_2_ showing the lowest scores. Flavor and juiciness, however, differed significantly (*p* < 0.05), with T_1_ and T_2_ outperforming T_0_. While these differences were statistically significant, the absolute numerical differences—particularly in flavor (0.6 points) and juiciness (1.0 point) on an 8-point scale—were modest and should be interpreted with caution when evaluating practical sensory benefits. Similar patterns were reported in previous studies, where flavor scores were highest in cinnamon-supplemented low-energy diets, while appearance and tenderness remained unaffected (18). The antioxidant properties of cinnamon (57) and turmeric (19) are known to enhance flavor stability. In the current study, T_1_ produced meat with the best flavor and juiciness, followed by T_2_, suggesting that energy restriction slightly attenuates the positive effects of vegetable waste supplemented with turmeric powder and cinnamon extract. Texture, appearance, and overall acceptability remained stable, indicating that the dietary modifications mainly influenced taste-related attributes rather than physical texture.

### 4.6 Liver enzyme profile

Despite a significant increase in liver weight observed in the T_1_ group, serum ALT and AST activities remained comparable across all treatments and within established physiological ranges, indicating no hepatocellular damage or leakage. This suggests that the increased liver weight likely reflects an adaptive metabolic response to the inclusion of 15% vegetable waste supplemented with cinnamon (0.1%) and turmeric (0.3%) in the diet, rather than pathological enlargement. These findings align with previous studies reporting that cinnamon supplementation supports liver health in broilers without adverse effects (58, 59). Moreover, the combination of vegetable waste and phytogenic additives appears to maintain normal liver function and overall broiler health.

### 4.7 Economic efficiency

Although the inclusion of PFA slightly increased feed cost, T1 achieved the highest gross profit percentage due to improved feed conversion efficiency, resulting in superior economic returns compared to the T0 (control) group.

Based on the economic analysis, T1—formulated with vegetable waste supplemented with turmeric powder and cinnamon extract while maintaining ME levels as per ICAR (2013) standards—was the most economically efficient treatment, supported by better feed conversion ratio, higher sale returns, greater gross profit, and superior production efficiency, consistent with earlier findings where cinnamon supplementation enhanced benefit–cost ratio (48) and turmeric inclusion improved profitability in broiler diets (37). T_2_, while benefiting from reduced feed cost through an approximately 10% lower ME diet, showed moderate performance and reduced profitability due to lower feed efficiency, whereas T0, relying solely on conventional feed ingredients, recorded the least favorable outcomes.

## 5 Conclusion

Incorporating 15% vegetable waste supplemented with 0.1% cinnamon extract and 0.3% turmeric powder significantly improved growth performance, nutrient digestibility, carcass yield, and economic efficiency in broilers without compromising meat quality. Maintaining ME levels as per ICAR (2013) standards was crucial to maximize these benefits, as excessive energy reduction limited performance. This feeding strategy provides a sustainable and cost-effective approach for broiler production. Future research should explore the long-term effects of such dietary inclusion on gut health and nutrient metabolism. Although the study used five replicates per treatment, the observed differences were associated with very large effect sizes (Cohen's *d* up to 5.30), supporting the adequacy of the replication level for detecting meaningful improvements in broiler performance.

## Data Availability

The original contributions presented in the study are included in the article/supplementary material, further inquiries can be directed to the corresponding author.
